# The Role of Slow Wave Sleep in Memory Pathophysiology: Focus on Post-traumatic Stress Disorder and Eye Movement Desensitization and Reprocessing

**DOI:** 10.3389/fpsyg.2017.02050

**Published:** 2017-11-22

**Authors:** Sara Carletto, Thomas Borsato, Marco Pagani

**Affiliations:** ^1^Department of Clinical and Biological Sciences, University of Turin, Turin, Italy; ^2^Department of Psychology, University of Milano-Bicocca, Milan, Italy; ^3^Institute of Cognitive Sciences and Technologies, National Research Council, Rome, Italy

**Keywords:** slow wave sleep, memory, EMDR, PTSD, cerebellum

Post-traumatic stress disorder (PTSD) is a clinical condition that may develop after a person experienced a traumatic event. PTSD can be considered as a disorder in which a fear conditioned response fails to extinguish, leading to several symptoms such as re-experiencing of the traumatic moment with intrusive thoughts, flashbacks, and nightmares, avoidance of situations related to the trauma, negative alterations in cognitions and mood, and hyper-arousal.

One important feature of PTSD is the re-experiencing of specific aspects of the traumatic memory. This aspect is related to the fact that, as originally suggested by Van Der Kolk et al. (1997); Van Der Kolk (1998), traumatic memories are encoded differently than memories of ordinary events, including several multisensory fragments that cannot be integrated in a structured meaningful narrative.

At a neurobiological level, memories recorded during extreme stressful situations cause a maximal potentiation of amygdalar synapses, assumed to temporarily store the events. This causes the saturation of all amygdalar alpha-amino-3-hydroxy-5-methyl-4-isoxazole (AMPA) receptors-bindings sites, preventing the recorded emotional memory trace to be merged with the cognitive memory trace from the hippocampus (Corrigan, 2002; Harper et al., 2009). Therefore, the fragments of emotionally charged memories remain trapped in the limbic system and cannot be transfered to the cortical areas, where a further processing and integration into already existing networks should take place.

Eye Movement Desensitization and Reprocessing (EMDR) was initially proposed in the late 80's by Francine Shapiro (Shapiro, 1989) and it's now a well-established psychological treatment for PTSD (Bradley et al., 2005; Chen et al., 2014). EMDR is a complex therapeutic approach that integrates elements of many traditional psychological orientations, but one of the key aspects is the use of Alternate Bilateral Stimulations (ABS) such as eye movements (EMs). Over recent years a large debate has developed whether EMs are an active treatment component and if the mechanisms responsible for EMDR efficacy differ substantially from those operating in trauma-focused cognitive behavioral therapy and standard exposure therapy. The original theory of Adaptive Information Processing (AIP), which is the basis of EMDR (Shapiro, 2001), posits that humans have an innate information processing system that assimilates new experiences into already existing memory networks. Pathology arises when new information is inadequately processed and then stored in a maladaptive mode in the memory networks, along with associated distorted thoughts, sensations and emotions. When memories are adequately processed, symptoms can be alleviated and memories integrated. EMDR seems to facilitate the access to and processing of the components of traumatic memories bringing them to an adaptive resolution (Shapiro, 2001). EMDR therefore aims at re-elaborating non-integrated memories and consolidating new memories into already existing semantic links, promoting the insertion of traumatic but no longer disturbing memory in a coherent and adaptive autobiography.

In recent years, the crucial role of sleep in memory consolidation has been highlighted (Stickgold and Walker, 2007; Rasch and Born, 2013). Physiological normal sleep presents a cyclic alternated pattern of Rapid Eye Movement (REM) and non-REM Slow Wave Sleep (SWS). EEG recordings show synchronous delta wave activity (0.5–4 cycles/sec, i.e., 0.5–4 Hz) during SWS, and synchronous theta waves (4–8 Hz) during REM sleep. The non-REM SWS appears to play a key role in memory consolidation, as edited memories are transferred from the hippocampus to the neocortex, and then integrated into neocortical neuronal networks upon this phase.

During the waking state, new memories are encoded in a temporarily form in the hippocampal network. Over SWS, such hippocampal memories are reactivated by slow oscillations (< 1 Hz) originating from the cortical networks in which the encoding originally took place (Born et al., 2006).

The combined activation of hippocampal memories and cortical synapses favors the transfer back to the neo-cortex, and during REM sleep the memories might be further consolidated and integrated in already existing associative links, therefore promoting a meaningful narrative of the event.

As mentioned above, traumatic memories may cause over-potentiation of amygdalar synapses, while the recording of the episodic aspect of the memory in the hippocampus results in a normal potentiation of hippocampal synapses. This difference in potentiation between amygdalar and hippocampal synapses makes impossible to merge the emotional and cognitive aspects of the traumatic memory, which normally takes place *via* the anterior cingulated cortex, then preventing the subsequent transfer to neocortex (Harper et al., 2009). Therefore, non-processed emotional memories remain trapped and unchanged at subcortical level without contextual integration, causing in some cases PTSD symptoms.

The first who proposed a similarity between EMDR physiological effects and sleep processes was Stickgold (2002), focusing mainly on REM sleep. Moving a step forward, one of the hypothesized mechanism of action of EMDR posits a parallel between synaptic depotentiation occurring during SWS and EMDR bilateral stimulation (Harper et al., 2009; Pagani et al., 2017). During EMDR the client is asked to hold into attention the fragmented traumatic materials while the therapist performs the ABS, favoring the reactivation of the traumatic memory in amygdalar and hippocampal networks. Recent findings have shown that ABS elicits delta waves in a range (1.5 Hz) very similar to the one registered during SWS (Rétey et al., 2005; Harper et al., 2009; Pagani et al., 2011, 2012). It is then conceivable that EMDR bilateral stimulation mimics the low-frequency stimulations naturally occurring during SWS, causing a depotentation of AMPA receptors of amygdalar synapses, which in turn facilitates the merging of the amygdalar emotional and the hippocampal episodic memories thus creating an associative memory that can be further transferred and processed by neocortical areas, leading to the cessation of symptoms.

This hypothesis on EMDR mechanism of action is also supported by previous neuroimaging findings. Pagani et al. (2012) showed a shift of cortical activation elicited by traumatic memories after successful EMDR treatment from an implicit subcortical to an explicit cortical state suggesting their integration into existing semantic memory. Herkt et al. (2014) observed an increased activation in the right amygdala during auditory ABS upon processing of negative emotional stimuli, suggesting that an initial enhancement of emotional processing is a prerequisite for the effective reintegration of traumatic material.

Other neurobiological findings frequently reported by neuroimaging studies in PTSD are supporting this hypothesis. Three main brain areas have been identify to be altered in PTSD: amygdala, involved in emotional interpretation of incoming information (Etkin and Wager, 2007; Francati et al., 2007; Patel et al., 2012), medial prefrontal cortex (mPFC), implicated in the processing of emotional materials and in emotion regulation (Shin et al., 2006; Patel et al., 2012; Nicholson et al., 2017) and hippocampus, involved in contextual learning and spatial and episodic memory (Burgess et al., 2002; Liberzon and Sripada, 2008; Liberzon and Abelson, 2016). The most recurrent finding is a relative decreased mPFC activity and a parallel increased amygdalar activation. In light of the SWS hypothesis such hyperactivation could be considered as the macroscopic expression of the molecular-level alteration, i.e., the above described over-potentiation of amygdalar synapses. At the same time mPFC, despite its tight connection with the limbic system, doesn't exert its role of normalizing amygdalar hyperactivity, probably as a consequence of the remarkable hyper-activation of the latter. The insufficiency of top-down prefrontal cortex functioning, coupled with dysregulated bottom-up activity of limbic structures accounts as well for the impaired autonomic response and concomitant inadequate emotional regulation peculiar of PTSD.

Recently, a pathologic bottom up mechanism involving cerebellum in PTSD has also been proposed (Carletto and Borsato, 2017). This structure has a key role in associative learning, fear regulation, attention, and in motor control (Wolf et al., 2009; Schmahmann, 2010) and it is strongly interconnected to cerebral cortex (Bergmann, 2008). Growing evidence shows altered functions of cerebellum in PTSD patients (Osuch et al., 2001; Anderson et al., 2002; Pissiota et al., 2002), with traumatic reminders ehnancing its activation (Fernandez et al., 2001; Driessen et al., 2004). Furthermore, cerebellar volume is smaller in adults with PTSD than in healthy controls (De Bellis and Kuchibhatla, 2006; Carrion et al., 2009; Baldaçara et al., 2011), and this reduction is associated with the magnitude of PTSD symptoms.

Cerebellum has also an indirect link to PTSD since it is a key structure in associative learning. In fact, PTSD can be described as the result of a fear conditioned response where extinction fails (Milad et al., 2009; VanElzakker et al., 2014). More specifically it is a conditioning to the context rather than to a specific stimulus (Davis et al., 2010) and it is well-documented how cerebellum appears to be involved in both conditioning and extinction process (Sacchetti et al., 2005; Kim and Jung, 2006). In a rodent study, Sacchetti et al. (2007) showed how cerebellum could be as crucial as amygdala in long term fear memories: when amygdala is inactivated the fear-conditioning response is carried out by cerebellum.

Zhu et al. (2006) analyzed the extensive connectivity between cerebellum and whole brain suggesting that this area is an essential modulator and coordinator of visceral and behavioral response through cerebellar-hypothalamic circuit.

Cerebellum has also a central role in the sleep-wake cycle (Cunchillos and De Andrés, 1982; de Andrés et al., 2011; DelRosso and Hoque, 2014). There seem to be a bidirectional interaction between cerebellum and sleep: malfunction of cerebellum affects quality of sleep but also quality of sleep affects cerebellum dependent memory formation and memory consolidation (Canto et al., 2017). Patients with spinocerebellar ataxia, a degenerating pathology of cerebellum and its connections, show alterations in sleep stages as well in non-wakefullness. In this way cerebellum tunes neocortical forms of sleep related activity (Pedroso et al., 2011; Canto et al., 2017). On the other hand, sleep improves cerebellar learning as learning-dependent timing, procedural memory formation, and spatiotemporal predictions of motor actions (Verweij et al., 2016).

Fogel et al. (2015) reported that sleep after a procedural task showed an increment in SWS density, highlighting a putative cerebellar involvement.

Typically, cerebellar activity decreases during the transition from pre-sleep wakefulness to SWS (Braun et al., 1997; Hofle et al., 1997; Kajimura et al., 1999; Hiroki, 2005). During SWS cerebellar fMRI signals co-occurred with slow waves in neo cortex, and the level of density of those waves in cerebellum was correlated with gray matter volume (Dang-Vu et al., 2008; Saletin et al., 2013).

According to Born et al. (2006) since the synchronization caused by slow oscillations during SWS is not restricted to neocortex but spreads to hippocampus, thalamus, and brainstem, it can be speculated that also cerebellum is involved in such synchronous activation which in turn might favor its involvement in processing the procedural aspect of the psychological trauma, an essential component of therapy successful outcome.

The role of cerebellum in such process needs further investigations: cerebellar transcranial Direct Current Stimulation (tDCS), a form of brain polarization that influences motor functions and learning processes (Gandiga et al., 2006) could be a promising tool in understanding this complex relation. Previous findings have shown that tDCS applied to frontocortical regions induced an increase in SWS and enhanced consolidation of hippocampal-dependent declarative memories (Marshall et al., 2004, 2006). Moreover, a recent study (Ferrucci et al., 2013) indicated that cerebellar tDCS improves implicit procedural learning. This technique could be potentially coupled with Electroencephalography, assessing brain frequencies evoked by this manipulation, in particular testing if cerebellar electrical field modulation could lead to a “SWS-like” brain activity.

In conclusion, this opinion article aims at underlining the role of SWS in the physiological storage of memories and its participation in putative mechanisms of recover from conditions in which memory remains pathologically unprocessed, as in PTSD. This will also stimulate further research, both at theoretical and experimental levels, on the role of various structures in formation, consolidation, and reprocessing of traumatic memories.

In our opinion, there is a need of deepening the current model of PTSD going beyond the role of top-down processes. Investigating other brain regions such as cerebellum in bottom-up processes involved in PTSD will allow to better understand the underlying mechanisms of this disorder and to promote effective and neurobiologically-grounded therapies for trauma treatment.

## Author contributions

MP and TB were responsible for the conception of the hypothesis outlined. SC wrote the article, that was integrated and critically revised by MP and TB. All authors have approved the final manuscript.

### Conflict of interest statement

All authors have been invited as speakers in national and international EMDR conferences. The authors declare that the research was conducted in the absence of any commercial or financial relationships that could be construed as a potential conflict of interest.
